# Androgen Receptor Expression in Early Triple-Negative Breast Cancer: Clinical Significance and Prognostic Associations

**DOI:** 10.3390/cancers6031351

**Published:** 2014-06-27

**Authors:** Mirco Pistelli, Miriam Caramanti, Tommasina Biscotti, Alfredo Santinelli, Alessandra Pagliacci, Mariagrazia De Lisa, Zelmira Ballatore, Francesca Ridolfi, Elena Maccaroni, Raffaella Bracci, Rossana Berardi, Nicola Battelli, Stefano Cascinu

**Affiliations:** 1 Clinica di Oncologia Medica, AO Ospedali Riuniti-Ancona, Università Politecnica delle Marche, Ancona 60020, Italy; 2 Anatomia Patologica, AO Ospedali Riuniti-Ancona, Università Politecnica delle Marche, Ancona 60020, Italy

**Keywords:** androgen receptor, triple negative, breast cancer, prognosis

## Abstract

*Background*: Triple-negative breast cancers (TNBC) are characterized by aggressive tumour biology resulting in a poor prognosis. Androgen receptor (AR) is one of newly emerging biomarker in TNBC. In recent years, ARs have been demonstrated to play an important role in the genesis and in the development of breast cancer, although their prognostic role is still debated. In the present study, we explored the correlation of AR expression with clinical, pathological and molecular features and its impact on prognosis in early TNBC. *Patients and Methods*: ARs were considered positive in case of tumors with >10% nuclear-stained. Survival distribution was estimated by the Kaplan Meier method. The univariate and multivariate analyses were performed. The difference among variables were calculated by chi-square test. *Results*: 81 TNBC patients diagnosed between January 2006 and December 2011 were included in the analysis. Slides were stained immunohistochemically for estrogen and progesterone receptors, HER-2, Ki-67, ALDH1, e-cadherin and AR. Of the 81 TNBC samples, 18.8% showed positive immunostaining for AR, 23.5% and 44.4% of patients were negative for e-cadherin and ALDH1, respectively. Positive AR immunostaining was inversely correlated with a higher Ki-67 (*p* < 0.0001) and a lympho-vascular invasion (*p* = 0.01), but no other variables. Univariate survival analysis revealed that AR expression was not associated with disease-free survival (*p* = 0.72) or overall survival (*p* = 0.93). *Conclusions*: The expression of AR is associated with some biological features of TNBC, such as Ki-67 and lympho-vascular invasion; nevertheless the prognostic significance of AR was not documented in our analysis. However, since ARs are expressed in a significant number of TNBC, prospective studies in order to determine the biological mechanisms and their potential role as novel treatment target.

## 1. Introduction

Triple negative breast cancer (TBNC) is a subtype of breast cancer defined by estrogen receptor (ER), progesterone receptor (PR) and human epidermal growth factor receptor 2 (HER2) negativity. Therefore it cannot benefit from endocrine therapy and HER-2-target therapy. 

Owing to the aggressive tumor biology and lack of targeted therapy, TNBC is characterized by a dismal although heterogeneous outcome. Recently, considerable efforts have been made to sub-classify TNBC into different prognostic groups to select patients who are candidates for more or less aggressive therapy regimens. Many studies have assessed new biomarkers able to identify patients with different prognosis. One of the most extensively investigated but also controversial biomarker is the androgen receptor (AR). Androgens are involved in the functions of different female organs, including the reproductive tract, bone, kidneys and muscle, acting indirectly as pro-hormones of estradiol or directly by binding to the androgen receptor (AR) [1]. The AR is the most prevalent sex steroid receptor occurring in up to 90% of breast cancers in early and metastatic setting [2,3,4] and in a lower rate in TNBC (0–53%) [5,6]. Although there is a growing evidence about the role of androgens and AR in breast cancer pathogenesis, the role of AR pathway in TNBC is still uncertain; conflicting results are reported in preclinical studies and their impact on clinical outcome is still debated [7,8,9]. In cell lines experiments androgens have been shown to have inhibitory and stimulatory effects on TNBC cell proliferation [10,11]. Several studies explored the potential significance of AR for therapeutic management of both primary and advanced disease, especially in TNBC due to the lack of any other targets. In the present study, we assessed ARs expression and their effect on prognosis in TNBC. 

## 2. Experimental

Breast cancer patients diagnosed with stage I–III TNBC, undergoing adjuvant chemotherapy at our Institution from January 2006 and December 2011, were eligible for this analysis. Patients who received preoperative chemotherapy or with stage IV of disease were excluded. We analyzed several parameters: clinical (age, performance status, type of surgery, adjuvant chemotherapy), pathological (tumor size, grading, necrosis, lymph nodes status, tumor histology, Ki-67, lympho-vascular invasion, androgen receptor expression) and molecular (ALDH1 and e-cadherin). 

### 2.1. Immunohistochemistry

Tissue samples were fixed in 10% buffered formalin and embedded in paraffin wax for routine histological examinations. The slides were stained with hematoxylin and eosin (H & E) with additional immunostaining for ER (clone: SP1, dilution: 1:200; NeoMarkers, Fremont, CA, USA), PR (clone: SP2, dilution: 1:250; NeoMarkers), Her2/neu (Herceptest, Dako, Carpinteria, CA, USA), Ki-67 (1:200, M7240, Dako), AR (1:60 F39.4.1, BioGenex San Ramon, CA, USA), ALDH1 (Clone 44/ALDH, 1:200, Transduction Laboratories, Franklin Lakes, NJ, USA) and e-cadherin (clone NCH-38, 1:50 Dako). ER, PR and AR were considered positive if there were at least 10% positive invasive tumor nuclei in the sample. HER-2 status was evaluated by immunohistochemistry (IHC) using a semiquantitative score (0–3+). Tumor staining was compared to the staining of normal breast epithelium from the same patient as a negative control. For clinical purposes, no staining or weak (1+) and incomplete membrans’staining was considered a negative result. Patients with 2+ IHC staining for HER2 underwent fluorescence in-situ hybridization to confirm HER2 negativty. Triple-negative status (ER negative, PR negative and HER-2 negative) was finally diagnosed and re-reviewed by the single study pathologist of our Institution. Carcinoma cells with cytoplasmic staining were considered to be ALDH1-positive cells. Any proportion of ALDH1-positive carcinoma cells was considered to represent epithelial ALDH1 expression. E-cadherin expression was semi-quantitative analysed according to the percentage of cells showing membrane positivity: 0, 0% to 10%; 1+, 10% to 30%; 2+ 30% to 70%; 3+ > 70%. E-cadherin expression was considered positive when scores were ≥2 and negative when scores were ≤1. 

### 2.2. Statistical Analysis

Disease-free survival (DFS) was defined as the interval between the date of diagnosis of TNBC to the date of relapse or progression of disease, or the date of death from any cause. Overall survival (OS) was defined as the interval between the date of diagnosis of TNBC to death or last follow-up visit. Patients who were not reported to be dead at the time of the analysis were censored at the date they were last known to be alive. Survival distribution was estimated by the Kaplan Meier method. The association between categorical variables was estimated by Chisquare test. The Cox multivariate proportional hazard regression model was used to evaluate the effects of the prognostic factors on survival. Significant differences in probability of surviving between the strata were evaluated by log-rank test. Hazard ratios and 95% confidence intervals (CIs) were estimated from regression coefficients. A significance level of 0.05 was chosen to assess the statistical significance. Statistical analysis was performed with MedCalc package (MedCalc® v9.4.2.0 Software, Ostend, Belgium).

## 3. Results

Eighty one (81) patients were included in the analysis. All patients were female, with a median age of 54 years (range 28–79 years). The majority of them (59.4%) had a menopausal status. Sixty-eight (83.8%) were treated with quadrantectomy and radiotherapy, 13 (16.2%) with radical mastectomy. Almost all patients (97.6%) received systemic adjuvant chemotherapy. Regimens are summarized in Table 1. Out of 81 TNBC, 76 (93.8%) were histologically identified as invasive ductal carcinoma. Major part of tumours (82.4%) showed an higher Ki-67 expression (>30%). Vascular invasion was found in 17 (21%) patients while the presence of necrosis was detected only in 7 cases. The median follow-up time was 52.4 months (range = 2.5–95 months). The median DFS was 44.8 months (range 2.5–95 months). Fifteen patients (18.8%) showed positive immunostaining for AR while 55.6%, and 76.5% were positive for ALDH1 and e-cadherin, respectively. Characteristics of 81 TNBC examined in the study are summarized in Table 1. Analysis of the relationship between AR immunostaining and clinico-pathological parameters revealed that positive immunostaining was inversely correlated with higher Ki-67 (*p* < 0.0001) and lympho-vascular invasion (*p* = 0.01). No significant difference between the groups with and without positive AR immunostaining in age at diagnosis, menopausal status, size of tumor, histological features, ALDH1 and e-cadherin immunostaining was identified (Table 1). 

**Table 1:** Baseline characteristics of the 81 patients with early TNBC.

Characteristics	Total No. of Pt (%)	AR negative No. of Pt (%)	AR positive No. of Pt (%)	*p*-value
Age				0.9
≤50 years	34 (42)	28 (34.6)	6 (7.4)	
>50 years	47 (58)	38 (46.6)	9 (11.4)	
Performance status ECOG 0 ECOG 1	67 (82.7) 14 (17.3)	57 (70.3) 9 (11.1)	10 (12.4) 5 (6.2)	0.68
Menopausal status Pre- Post-	33 (40.6) 48 (59.4)	27 (33.2) 39 (48)	6 (7.4) 9 (11.4)	0.8
Tumour size pT1 pT2-T4	46 (56.8) 35 (43.2)	36 (44.4) 30 (37)	10 (12.4) 5 (6.2)	0.5
Lymph node status (pN) pN0 pN+ pNx	46 (56.8) 31 (38.3) 4 (4.9)	38 (46.8) 24 (29.7) 4 (4.9)	8 (10) 7 (8.6) 0 (0)	0.5
Tumour histology Ductal carcinoma Lobular carcinoma Other	76 (93.8) 4 (4.8) 1 (1.2)	63 (77.6) 2 (2.4) 1 (1.2)	13 (16.2) 2 (2.4) 0 (0)	0.17
Histologic grade I–II III	6 (7.4) 75 (92.6)	3 (3.7) 63 (77.8)	3 (3.7) 12 (14.8)	0.1
Ki-67 ≤30% >30%	14 (17.6) 67 (82.4)	5 (6.2) 61 (75)	9 (11.4) 6 (7.4)	<**0.0001**
Lympho-vascular invasion Yes No	17 (21) 64 (79)	10 (12,4) 56 (69)	7 (8,6) 8 (10)	**0.01**
Lymphocytic infiltrate Yes No	13 (16) 68 (84)	12 (14.8) 54 (66.4)	1 (1.2) 14 (17.6)	0.4
Necrosis Yes No	7 (8.6) 74 (91.4)	5 (6.2) 61 (75.2)	2 (2.4) 13 (16.2)	0.8
Intraductal carcinoma Yes No	34 (42) 47 (58)	26 (32) 40 (49.4)	8 (10) 7 (8.6)	0.4
Type of surgery Quadrantectomy Radical mastectomy	68 (83.8) 13 (16.2)	57 (70.3) 9 (11.4)	11 (13.5) 4 (4.8)	0.31
Adjuvant chemotherapy Antracycline containing CMF No	47 (58.1) 32 (39.5) 2 (2.4)	39 (48.1) 26 (32.1) 1 (1.2)	8 (10) 6 (7.4) 1 (1.2)	0.51
ALDH1 Positive Negative	45 (55.6) 36 (44.4)	39 (48.2) 27 (33)	6 (7.4) 9 (11.4)	0.2
E-cadherin Positive Negative	62 (76.5) 19 (23.5)	50 (61.7) 16 (19.8)	12 (14.8) 3 (3.7)	0.9
Recurrences Yes No	16 (19.9) 65 (80.1)	13 (16.2) 53 (65.3)	3 (3.7) 12 (14.8)	0.7
Deaths Yes No	10 (12.4) 71 (87.6)	8 (10) 58 (71.4)	2 (2.4) 13 (16.2)	0.7

Abbreviations: AR = androgen receptor; CK 5/6 = cytokeratins 5/6; ALDH1 = aldehyde dehydrogenase 1.

Univariate survival analysis revealed that positive immunostaining for AR was not associated with DFS (*p* = 0.72) and OS (*p* = 0.93) (Table 2 and Table 3). No clinical or biological variables were significantly related to DFS while the only factor which is significantly related to a worsened OS was the presence of necrosis (*p* = 0.002). Multivariate analysis confirmed that the presence of necrosis was the significant independent prognostic variable influencing OS (*p* = 0.007; HR = 6.78, 95% CI 1.67–27.5). 

**Table 2:** Univariate Cox regression analysis of factors associated with disease-free survival in early triple negative breast cancer.

Parameters	Univariate analysis		
	HR	95% CI	*p*-value
Age, years ≤ge *vs.* >50	0.9	0.3–2.47	0.8
Menopausal status Pre- *vs.* post-	0.94	0.3–2.6	0.9
Tumour size (at diagnosis) pT1 *vs.* pT2-T4	0.7	0.2–198	0.5
Lymph node status (pN) pN0 *vs.* pN+	0.81	0.4–3.2	0.8
Histologic grade G1-G2 *vs.* G3	1.33	0.25–7.69	0.6
Ki-67 ≤i-6 *vs.* >30%	0.8	0.23–2.71	0.71
Necrosis negative *vs.* positive	0.31	0.01–1.01	0.05
Lympho-vascular invasion negative *vs.* positive	0.42	0.08–1.23	0.09
Lymphocytic infiltrate negative *vs.* positive	1.16	0.28–4.74	0.8
Intraductal carcinoma negative *vs.* positive	1.12	0.41–3.05	0.8
e-cadherin negative *vs.* positive	1.1	0.3–3.26	0.9
ALDH1 negative *vs.* positive	1.79	0.67–5.02	0.23
AR negative *vs.* positive	1.24	0.36–4.24	0.72

**Table 3:** Univariate and multivariate Cox regression analysis of factors associated with overall-survival in early triple negative breast cancer.

Parameters	Univariate analysis			Multivariate analysis		
	HR	95% CI	*p*-value	HR	95% CI	*p*-value
Age, years ≤ge *vs.* >50	0.6	0.18–2.28	0.49	-		
Menopausal status Pre- *vs.* post-	0.6	0.18–2.33	0.51	-		
Tumour size (at diagnosis) pT1 *vs.* pT2-T4	0.48	0.13–1.69	0.25	-		
Lymph node status (pN) pN0 *vs.* pN+	0.7	0.4–2.9	0.43	-		
Histologic grade G1-G2 *vs.* G3	2.52	0.43–33.6	0.22	-		
Ki-67 ≤i-6 *vs.* >30%	1	0.21–4.73	0.99	-		
Necrosis negative *vs.* positive	2.15	1.12–3.23	**0.002**	6.78	1.67–27.5	**0.007**
Lympho-vascular invasion negative *vs.* positive	0.31	0.03–1.04	0.056	-		
Lymphocytic infiltrate negative *vs.* positive	1.7	0.28–8.47	0.6	-		
Intraductal carcinoma negative *vs.* positive	1.55	0.44–5.62	0.48	-		
e-cadherin Negative *vs.* positive	1.62	0.36–8.05	0.48	-		
ALDH1 Negative *vs.* positive	3.27	0.91–11.3	0.06	-		
AR Negative *vs.* positive	0.93	0.19–4.56	0.93	-		

## 4. Discussion

TNBC represents a group of breast cancer with poor prognosis, owing to aggressive tumor biology and lack of targeted therapy-like HER2 blocking agents or hormonal therapy [12]. However, several reports suggested that TNBC could represent a heterogeneous group comprising different subtypes with different clinical outcomes. Many published studies have attempted to identify new biomarkers to combine with those available in clinical practice to sub-classify TNBC into different prognostic groups and to select patients who are candidates for more aggressive therapy regimens.

There is a growing body of evidence that androgen signaling pathway plays a critical role in normal and malignant breast tissue [13]. The AR is the most prevalent sex steroid receptor occurring in up to 90% of all breast cancers (*in situ* and invasive disease including early and metastatic setting) [2,3,4] and in a lower rate in TNBC (0%–53%) [5,6]. The overall frequency of AR in carcinoma cells varies considerably among the studies, even if a 10% cut-off point has been selected to define AR positivity. This heterogeneity may be largely due to the methodological differences in AR analysis. Further, several analyses based on unselected breast cancer cohorts showed that AR expression is related with ERα and PgR immunostaining as well as a marker of low grade differentiated disease [2,3,4,14,15,16]. Although several pre-clinical and genomic studies defined AR as a potential tumor suppressor of ERα-positive breast cancer with antiproliferative effect due to the cross talk between these steroid receptor signaling pathways [4], studies investigating the biological role and the clinic-pathological features of AR expression in TNBC reported conflicting results. Furthermore, Farmer *et al*. [17] proposed a new classification of breast cancer subtypes based on ERα and AR status: luminal disease positive for both receptors (ERα+/AR+), basal disease negative for both receptors (ERα−/AR−) and molecular apocrine disease with ERα negative and AR positive staining, showing similar characteristics to those of the normal apocrine glands. Following analyses showed that in molecular apocrine cancers there is a cross talk between AR and HER2 pathway with a trend of poor outcome [18,19,20]. While ARs showed inhibitory activity on ERα pathway with antiproliferative effect in ERα-positive breast tumor cells and ARs levels could be predictive of the outcome in luminal subtypes [21,22], their role is still unclear in TNBC. 

Birell *et al*.’s [10] *in vitro* analyses indicated a growth inhibition effect of AR pathway in cell lines expressing ER and PR, while proliferative effects were found in ER and PR negative cell lines. The authors suggested that androgen action may be mediated, beyond by binding of androgen to the androgen receptors, by metabolites of dihydrotestosterone (DHT), also due to its estrogenic activity. Garay *et al*. [11] reported that the activation of the mitogen activated protein kinase (MAPK) pathway by either EGFR or AR leads to cellular proliferation, but they also found that hyperactivation of the MAPK pathway from both AR and EGFR signaling resulted in a growth-inhibitory response. Furthermore some results of *in vitro* investigations suggested that AR may displace ER and PR as a driver of tumor proliferation and growth in TNBC cell lines [1,13,23,24,25,26,27,28]. Several published studies have analyzed the relationship between AR immunostaining and clinico-pathological parameters. The expression of AR was inversely correlated with higher clinical stage, higher mitotic score and higher histological grade suggesting AR positive TNBC could be less aggressive tumors [7,8,24,25,26,27,28,29,30,31]. On the contrary McGhan *et al*. [32] reported in their study a higher propensity for lymph node (LN) metastases, more advanced disease and poorer recurrence free survival in AR positive TNBC. In our study a positive immunostaining for AR was detected in 18.8% of TNBC.

AR positivity was inversely correlated with higher Ki-67 (*p* < 0.0001) and lympho-vascular invasion (*p* = 0.01), while we did not find any relationship with age, menopausal status, size of tumor, histological features, ALDH1 and e-cadherin immunostaining (Table 1). Similar findings were reported by Mrklic *et al*. [33]. They observed an inversely correlation between proliferative index measured by Ki 67 antigen and AR status (33.7% *vs*. 7.2%, *p* = 0.014), suggesting that androgens might have an antiproliferative effect. AR immunostaining was also inversely correlated with higher clinical stage (*p* = 0.0259, higher mitotic score (*p* = 0.003) and higher histological grade (*p* = 0.038), but no significant difference between two groups in vascular invasion and other parameters. Sutton *et al*. [34] revealed that among the AR-positive TNBC, distant metastases are significantly associated with lower expression of AR compared with cases with only locoregional disease, and that AR expression negatively correlates with Ki-67 expression. Other studies observed an inverse correlation between positive AR immunostaining and high Ki-67 status [7,31,35,36,37].

Several studies in recent years highlighted the role of AR as independent prognostic factor for luminal breast cancer, confirming their tumor suppressive effect on ERα pathway [4]. On the other hand the impact on outcome of AR expression in triple-negative tumors is still unclear. 

Some studies indicated an improved survival [5,6,32,33], while others worse survival [30] or no significant effects [13,18,25,26,27]. Our study did not show any significant correlation between AR status and survival. However, there are some limitations to this study. This is a retrospective analysis of data collected in a single institution, on a total number of patients included relatively small and so it could be underpowered to make definitive conclusions.

The relevant discordance about the prognostic role of AR expression may be due to the complexity of this pathway. McNamara *et al*. analyzed [35] the AR status in combination with androgen synthesizing enzyme 5α-reductase type 1 (5αR1) and 17β-hydroxysteroid dehydrogenase type 5 (17βHSD5). The presence of androgen synthesizing pathway in addition to AR expression in tumor cells (AR+/enzyme+) could confer a better clinical outcome through suppression of cell proliferation. Therefore, if we want to know the impact of AR expression on prognosis, we need to asses not only the receptor expression but also its ligand. Only in presence of a double positivity we could observe a better outcome. 

To date the AR is largely explored as potential therapeutic target, and androgen treatment showed some therapeutic benefit in addition to chemotherapy or Tamoxifen in several randomized trials [4,38,39,40,41,42,43]. Other interesting evidence are emerging particularly from androgen hormones treatment that may represent a new relevant therapeutic approach for TNBC expressing AR positivity. Promising results were obtained by Gucalp *et al*. [44] in a Phase II trial that showed clinical benefit rate of 19% for 6 months in 424 patients with ER/PgR-negative BC underwent bicalutamide 150 mg daily with minimal toxicity. These results could suggest that in some circumstances androgens may drive tumour proliferation. 

## 5. Conclusions

In conclusion, our study examined the relationship between AR expression and clinical and prognostic outcomes in an italian cohort of 81 patients with early TNBC. Univariate analysis revealed that AR positivity was inversely correlated with higher Ki-67 (*p* < 0.0001) and lympho-vascular invasion (*p* = 0.01), although in multivariate analysis this effect was not significant. These findings were in line with some of previously published studies. No significant correlation between AR status and survival was found. This data is of importance to a growing body of evidence documenting the association between AR expression and biological factors in TNBC. However, the role of AR pathway in TNBC is still uncertain; conflicting results are reported in preclinical studies and their impact on clinical outcome is still debated. Further prospective studies are needed to determine the pathogenetic mechanism underlying androgen and their receptors in TNBC to adequately assess the potential role of AR pathway as druggable target. 
